# Tryptophan metabolism and its relationship with central nervous system toxicity in people living with HIV switching from efavirenz to dolutegravir

**DOI:** 10.1007/s13365-018-0688-3

**Published:** 2018-11-26

**Authors:** Michael R. Keegan, Alan Winston, Chris Higgs, Dietmar Fuchs, Adriano Boasso, Mark Nelson

**Affiliations:** 1 HIV Research Unit, Clinical Trials Centre, Winston Churchill Wing, St Mary’s Hospital, Imperial College London, Praed Street, London, W2 1NY UK; 2 ViiV Healthcare Ltd., Brentford, UK; 3 Chelsea & Westminster Hospital, London, UK; 4 Innsbruck Medical University, Innsbruck, Austria

**Keywords:** HIV, Tryptophan, Kynurenine, Efavirenz, Dolutegravir, Central nervous system

## Abstract

The mechanisms underlying central nervous system (CNS) toxicities in antiretroviral-treated persons living with HIV (PLWH) remain elusive. We investigated the associations between markers of tryptophan metabolism and measurements of CNS toxicity in PLWH. In a prospective study, virologically suppressed PLWH receiving efavirenz-containing antiretroviral regimens with ongoing CNS toxicity were switched to dolutegravir-containing regimens and followed up for 12 weeks. Plasma tryptophan and kynurenine concentrations and the kynurenine/tryptophan ratio were calculated. Ten CNS toxicities were graded according to the ACTG adverse events scale. Scores ranged from 0 (none) to 3 (severe) and were summed, giving a total from 0 to 30. Paired-samples *t* tests and linear mixed model analyses were conducted to assess changes in, and relationships between, laboratory and clinical parameters. Mean kynurenine plasma concentration increased from baseline to week 12 (2.15 to 2.50 μmol/L, *p* = 0.041). No significant changes were observed for tryptophan (54.74 to 56.42 μmol/L, *p* = 1.000) or kynurenine/tryptophan ratio (40.37 to 41.08 μmol/L, *p* = 0.276). Mean CNS toxicity score decreased from 10.00 to 4.63 (*p* < 0.001). Plasma kynurenine concentration correlated with CNS toxicity score: for every 1 μmol/L increase in kynurenine concentration observed, a 1.7 point decrease was observed in CNS toxicity score (*p* < 0.038). A similar trend was observed for the kynurenine/tryptophan ratio: for every 1 μmol/mmol increase observed in kynurenine/tryptophan ratio, a 0.1 point decrease was observed in CNS toxicity score (*p* = 0.054). Switching from efavirenz to dolutegravir was associated with increases in plasma kynurenine concentration and improvements in CNS toxicity scores. Underlying mechanisms explaining the rise in kynurenine concentrations need to be established.

## Introduction

Many antiretroviral agents are associated with central nervous system (CNS) toxicities. Efavirenz is a widely used agent and is associated with CNS clinical toxicities including vivid dreams, insomnia, cognitive impairment, suicidal ideation and suicide (Summary of product characteristics, Sustiva® 2016; Cavalcante et al. 2010). This has resulted in HIV treatment guidelines moving towards the use of HIV-integrase-inhibitors as first line antiretroviral regimens (European AIDS Clinical Society (EACS) n.d.; British HIV Association (BHIVA) n.d.; United States Department of Health and Human Services (DHHS) n.d.; Gunthard et al. 2016). Although overt CNS side effects may be less prevalent with the integrase-inhibitor class, emerging toxicities are being reported (Fettiplace et al. 2016).

The pathogeneses of CNS toxicities from antiretroviral agents remains elusive (Underwood et al. 2015). One potential mechanism is direct neuronal toxicity. Efavirenz and its major metabolite, 8-hydroxy-efavirenz, have been shown to be toxic in neuron cultures at concentrations found in cerebrospinal fluid (Robertson et al. 2012; Tovar-y-Romo et al. 2012). Other potential mechanisms may include alterations to tryptophan metabolism. Tryptophan acts as a substrate for tryptophan 5-hydroxylase, leading to the production of 5-hydroxytryptophan (serotonin). Under physiological conditions, tryptophan is also degraded via hepatic metabolism by tryptophan 2,3-dioxygenase (TDO), which is activated when the concentration of tryptophan exceeds the requirements for its metabolic needs. Tryptophan is also catabolised extrahepatically in intestinal, lung, placenta and brain tissues by indoleamine 2,3-dioxygenase (IDO-1), which is inducible by proinflammatory cytokines (Stone 1993; Fuchs et al. 1990; Boasso and Shearer 2007). In the CNS, the cellular localization of IDO-1 has been shown to be primarily in infiltrating macrophages and resident microglial cells (Guillemin et al. 2003; Guillemin et al. 2004). Increasing IDO-1 activity diverts tryptophan away from serotonin production and results in the production of kynurenine and a number of neuroactive catabolites, most of which are either neurotoxic or neuroprotective (Stone 1993; Fuchs et al. 1990; Boasso and Shearer 2007; Guillemin et al. 2003; Guillemin et al. 2004). The net result during HIV infection is unclear, but there is potential for neural damage to occur, which could impact cognition and mood (Davies et al. 2010; Heyes et al. 1992; Keegan et al. 2016; Grill et al. n.d.).

The impact of antiretroviral therapy (ART) on tryptophan metabolism has yet to be fully explored. In PLWH, virologically suppressive ART has been shown to reduce, though not necessarily normalise, tryptophan metabolism. This is thought to be via the kynurenine pathway through its ability to reduce viral loads and attenuate immune activation (Zangerle et al. 2002; Byakwaga et al. 2014; Chen et al. 2014). However, previous studies have not assessed the potential differential effects of specific ART drugs. Efavirenz has been show to inhibit TDO activity in the liver cells of non-HIV-infected rats, but it is unclear whether this is associated with neurotoxicity (Zheve 2007).

Our aim was to explore the effects of switching from efavirenz to dolutegravir on tryptophan metabolism and CNS toxicity in virologically suppressed PLWH.

## Methods

### Study design

We conducted a prospective, randomised, open-label, multi-centre study, in which virologically suppressed PLWH receiving two nucleoside reverse transcriptase inhibitors (NRTIs) plus efavirenz for ≥ 12 weeks who had reported experiencing ongoing CNS toxicity were enrolled. Subjects were randomised to switch immediately to dolutegravir (*n* = 20) or have their switch delayed for 4 weeks (*n* = 20), without NRTI change. Both groups were followed up for a further 12 weeks. The main study results have been reported (Bracchi et al. n.d.). In this analysis, the study arms were pooled and ‘baseline’ was defined as the time of ART switch for all subjects.

### Clinical assessments

Participants received a comprehensive medical examination, which included an assessment of HIV disease (i.e. current and nadir CD4+ cell count, and plasma HIV-1 RNA) and ART characteristics.

Rates of CNS toxicities were measured at baseline, week 4 and week 12 using a questionnaire based on the efavirenz label and graded according to the ACTG adverse events scale. The questionnaire consisted of 10 sections specifically ascertaining the following symptoms: dizziness, depression, insomnia, anxiety, confusion, impaired concentration, headache, somnolence, aggression and abnormal dreams. Each score ranged from 0 (none) to 3 (severe) with scores summed, giving a total score ranging from 0 to 30.

### Laboratory methods

Plasma concentrations of tryptophan and kynurenine were measured by high-performance liquid chromatography (HPLC) using the ProStar 210 solvent delivery system (Agilent Technologies Inc.) (Laich et al. 2002). Sample injection was controlled by a ProStar 400 autosampler, a ProStar 360 fluorescence detector and a ProStar 325 ultraviolet detector (Agilent). Separation was accomplished at room temperature using a reversed-phase LiChroCART 55–4 mm cartridge (Merck), filled with Purospher STAR RP-18 (3 μm grain size; Merck) together with a reversed-phase C18 precolumn (Merck). Before HPLC, serum protein was precipitated with 0.015 mM trichloroacetic acid. For both measurements, L-nitro-tyrosine is used as an internal standard and monitored at the 360 nm wavelength.

Tryptophan and kynurenine concentrations were measured in one chromatographic run using dihydrogen phosphate solution for separation on reversed-phase C18 material with mobile phase 0.015 M sodium acetate/acetic acid (pH = 4) + 5% methanol and with the fluorescence detector set at 285 nm excitation and 360 nm emission wavelengths. Ultraviolet absorption to detect kynurenine and L-nitro-tyrosine concentrations was measured at the 360 nm wavelength (Laich et al. 2002; Widner et al. 1997). The kynurenine/tryptophan ratio was calculated, estimating IDO-1 activity.

Neopterin concentrations were measured by enzyme-linked immunosorbent assay (BRAHMS Diagnostics) following the manufacturer’s protocol (sensitivity, 2 nmol/L).

### Statistics

Statistical analyses were performed with IBM’s SPSS Software Version 23. The results were subjected to tests of normal distribution. The data were found to be normally distributed and so paired-samples *t* tests were conducted to assess changes from baseline to week 4, week 4 to week 12, and baseline to week 12 for tryptophan, kynurenine and neopterin concentrations, the kynurenine/tryptophan ratio, and CNS toxicity scores. To investigate the relationship between CNS toxicity scores and kynurenine concentrations and the kynurenine/tryptophan ratio, linear mixed models were constructed.

Bonferroni corrections were applied to each group of analyses to adjust the level of significance to account for multiplicity. Only the adjusted *p* values are shown.

## Results

### Subject characteristics

Most subjects were male (*n* = 38; 95%) and of white ethnicity (*n* = 38; 95%). Mean age was 48 years (standard deviation [SD] 11). All subjects were virologically suppressed (HIV-1-RNA < 50 copies/mL) at baseline and maintained suppression throughout the 12 weeks. Mean CD4+ cell count/μL was 604 (SD = 201) and 679 (SD = 219) at baseline and week 12, respectively. NRTI backbones were either tenofovir and emtricitabine (*n* = 39) or zidovudine and lamivudine (*n* = 1).

### Laboratory and clinical measurements

Mean plasma concentrations of tryptophan, kynurenine, neopterin and the kynurenine/tryptophan ratio are presented in Table 1. The mean plasma concentration of kynurenine increased significantly from baseline to week 12 (adjusted *p* = 0.041). Numerical increases were observed for the kynurenine/tryptophan ratio from baseline to week 12 (adjusted *p* = 0.456) and week 4 to week 12 (adjusted *p* = 0.276), but these were not statistically significant following Bonferroni correction. No significant changes were observed for tryptophan or neopterin at any time point (Table 1).

**Table 1:** Changes in laboratory and clinical parameters

Parameter, mean	Number	Baseline	Week 4	Week 12	Mean change					
					BL to Wk4	Adjusted *P* value	Wk4 to Wk12	Adjusted *P* value	BL to Wk12	Adjusted *P* value
TRP, μmol/L (SD)	36	54.74 (10.59)	57.38 (15.38)	56.42 (11.71)	2.63 (15.76)	1.000	− 0.96 (17.48)	1.000	1.67 (13.38)	1.000
KYN, μmol/L (SD)	36	2.15 (0.59)	2.29 (0.67)	2.50 (0.76)	0.14 (0.66)	1.000	0.21 (0.82)	1.000	0.35 (0.66)	*0.041*
KYN/TRP ratio, μmol/mmol (SD)	36	40.37 (12.48)	41.08 (10.94)	44.99 (12.41)	0.71 (7.55)	1.000	3.91 (10.91)	0.456	4.62 (11.67)	0.276
NEO, μmol/L (SD)	36	13.76 (9.29)	12.60 (7.42)	12.39 (6.09)	1.16 (7.13)	1.000	− 0.21 (6.34)	1.000	− 1.36 (6.95)	1.000
CNS toxicity (SD)	38	10.00 (4.69)	4.79 (4.07)	4.63 (4.24)	− 5.21	*< 0.001*	− 0.16 (2.38)	1.000	− 5.37 (4.73)	*< 0.001*

A*P* value of equal to or less than 0.05 is considered to be significant following Bonferroni correction for multiplicity

*TRP* tryptophan, *KYN* kynurenine, *NEO* neopterin, *CNS* central nervous system, *SD* standard deviation, *CI* confidence interval

A significant reduction was observed in the mean CNS toxicity score from baseline to week 4 (adjusted *p* < 0.001) and baseline to week 12 (adjusted *p* < 0.001), indicating improvement (Table 1). No significant change was observed between week 4 and week 12 (adjusted *p* = 1.000).

### Factors associated with markers of tryptophan metabolism

In the linear mixed model analyses (Table 2), plasma kynurenine concentrations were found to be negatively correlated with CNS toxicity scores, such that for every 1 μmol/L increase observed in kynurenine concentration, a 1.7 point decrease was observed in the CNS toxicity score (adjusted *p* = 0.038). Likewise, a trend was observed for the kynurenine/tryptophan ratio, such that for every 1 μmol/mmol increase observed in the kynurenine/tryptophan ratio, a 0.1 point decrease was observed in the CNS toxicity score (adjusted *p* = 0.054).

**Table 2:** Linear mixed model results for KYN and KYN/TRP ratio and CNS toxicity from baseline to week 12

Parameter	Estimate (95% CI)	Adjusted *P* value
Model 1: CNS toxicity and KYN		
Mean CNS toxicity score	10.4 (7.0 to 13.9)	*< 0.001*
KYN, μmol/L	− 1.7 (− 3.1 to − 0.3)	*0.038*
Model 2: CNS toxicity and KYN/TRP ratio		
Mean CNS toxicity score	10.4 (6.8 to 14.1)	*< 0.001*
KYN/TRP ratio, μmol/mmol	− 0.1 (− 0.2 to − 0.0)	0.054

*TRP* tryptophan, *KYN* kynurenine, *CNS* central nervous system, *CI* confidence interval

## Conclusions

In this study, virologically suppressed PLWH experiencing ongoing CNS toxicity with efavirenz were switched to dolutegravir, resulting in significant improvements in CNS adverse events. Following switch, a significant increase was observed in plasma kynurenine concentrations and this change was negatively correlated with the reduction in CNS toxicities.

The improvements in clinical parameters are expected. Several studies have demonstrated similar results when switching from efavirenz to other antiretroviral agents, such as etravirine, raltegravir and rilpivirine (Waters et al. 2011; Yapa et al. n.d.; Rowlands et al. n.d.). However, the increases in plasma kynurenine concentrations and the kynurenine/tryptophan ratio were surprising. Previous studies have demonstrated that kynurenine/tryptophan ratios decline following initiation of ART due to decreases in immune activation resulting from suppression of HIV-1-RNA (Zangerle et al. 2002; Byakwaga et al. 2014; Chen et al. 2014). In this study, neopterin was selected as a marker of immune activation based on its correlation with IDO activity (Fuchs et al. 1991). The lack of correlation between neopterin and kynurenine concentrations observed is consistent with the hypothesis that increases in kynurenine concentrations may be due to changes in hepatic tryptophan metabolism rather than immune-driven IDO activity. However, we only have one marker of immune activation (neopterin) and, therefore, cannot validate this hypothesis by assessing other markers of inflammation. Efavirenz is a potent inhibitor and inducer of cytochrome P450 enzymes in the liver (McDonagh et al. 2015). Unpublished data by Zheve showed that efavirenz inhibits TDO activity in the liver cells of non-HIV-infected rats (Zheve 2007). If this effect translates to human subjects, then removal of efavirenz would result in an increase in TDO activity and an increase in kynurenine production, as we observed. Whilst this is a plausible explanation, we cannot exclude other immunologic or virologic factors that may have influenced tryptophan metabolism and kynurenine production and remain undetected in our study.

The results of the mixed model analyses suggest that the improvements in CNS toxicity scores are associated with the increases in kynurenine concentrations. Kynurenic acid, a downstream catabolite of kynurenine, is known to be neuroprotective due to its ability to block excitotoxic neuronal damage (Foster et al. 1984; Andine et al. 1988) and has been shown to be elevated in the brains of PLWH (Baran et al. 2000). Whilst we did not measure changes in this compound, it is interesting to speculate that increases in kynurenic acid concentration or activity following the removal of efavirenz could be attenuating neuronal damage and contributing to the improvements in CNS toxicity. It is important to note that other mechanisms could also be contributing. For example, the direct neurotoxic effects of the 8-hydroxy-efavirenz metabolite were not measured in this study and it is possible that they may be confounding our observations. Our data should be interpreted with caution.

There are some important limitations to our study. Firstly, we only measured tryptophan and kynurenine concentrations in plasma. Cerebrospinal fluid analysis would be useful to help determine whether there are similar changes occurring in the CNS compartment. In addition to this, analysis of other kynurenine pathway catabolites, such as kynurenic acid and quinolinic acid, as well as additional markers of immune activation, would be useful to help explain our observations. Measurement of serotonin concentrations, which are known to be lower in the blood and cerebrospinal fluid of PLWH, would also be helpful (Launay et al. 1988; Larsson et al. 1989).

Another limitation is the open-label nature of the study and the subjective nature of the CNS toxicity scoring questionnaire, which may have introduced bias.

Given that most of our participants were white males, these findings may not be extrapolatable to other ethnicities or females. Differences in TDO activity have been reported in men and women of various ethnicities (Badawy and Dougherty 2016). There are six reported IDO-1 genetic transcript variants, the effects of which are currently unknown (Murray 2007).

Lastly, we did not assess the potential contribution of dietary differences to tryptophan concentration (Strasser et al. 2016).

In summary, switching from efavirenz to dolutegravir resulted in changes in tryptophan metabolism and improvements in measures of CNS toxicity. Future studies to confirm and expand on our observations and elucidate the underlying pathogenic mechanisms are warranted.

## References

[CR1] Andine P, Lehmann A, Ellren K (1988). The excitatory amino acid antagonist kynurenic acid administered after hypoxic-ischemia in neonatal rats offers neuroprotection. Neurosci Lett.

[CR2] Badawy AAB, Dougherty DM (2016). Assessment of the human kynurenine pathway: comparisons and clinical implications of ethnic and gender differences in plasma tryptophan, kynurenine metabolites, and enzyme expressions at baseline and after acute tryptophan loading and depletion. Int J Tryptophan Res.

[CR3] Baran H, Hainfellner JA, Kepplinger B, Mazal PR, Schmid H, Budka H (2000). Kynurenic acid metabolism in the brain of HIV-1 infected patients. J Neural Transm.

[CR4] Boasso A, Shearer GM (2007). How does indoleamine 2,3-dioxygenase contribute to HIV-mediated immune dysregulation. Curr Drug Metab.

[CR5] Bracchi M, Pagani N, Clarke A, Adams T, Waters L, Bolton M, et al (n.d.) Multicentre open-label pilot study of switching from efavirenz to dolutegravir for central nervous system toxicity. International Congress of Drug Therapy in HIV Infection 23-26 October 2016, Glasgow, UK, p 209

[CR6] British HIV Association (BHIVA) (n.d.) Guidelines for the treatment of hiv-1-positive adults with antiretroviral therapy 2015 (2016 Interim Update). http://www.bhiva.org/HIV-1-treatment-guidelines.aspx 10.1111/hiv.12426 27568911

[CR7] Byakwaga H, Boum Y, Huang Y, Muzoora C, Kembabazi A, Weiser SD (2014). The kynurenine pathway of tryptophan catabolism, CD4+ T-cell recovery, and mortality among HIV-infected Ugandans initiating antiretroviral therapy. J Infect Dis.

[CR8] Cavalcante GIT, Capistrano VLM, Cavalcante FSD, Vasconcelos DS, Macedo DS, Sousa FCF (2010). Implications of efavirenz for neuropsychiatry: a review. Int J Neurosci.

[CR9] Chen J, Shao J, Cai R, Shen Y, Zhang R, Liu L, Qi T, Lu H (2014). Anti-retroviral therapy decreases but does not normalize indoleamine 2,3-dioxygenase activity in HIV-infected patients. PLoS One.

[CR10] Davies NWS, Guillemin G, Brew BJ (2010). Tryptophan, neurodegeneration and HIV-associated neurocognitive disorder. Int J Tryptophan Res.

[CR11] European AIDS Clinical Society (EACS) (n.d.) Guidelines Version 8.1, October 2016. http://www.eacsociety.org/files/guidelines_8.1-english.pdf

[CR12] Fettiplace A, Stainsby C, Winston A, Givens N, Puccini S, Vannappagari V (2016). Psychiatric symptoms in patients receiving dolutegravir. J Acquir Immune Defic Syndr.

[CR13] Foster AC, Vezzani A, Frencg ED, Schwarz R (1984). Kynurenic acid blocks neurotoxicity and seizures induced in rats by the related brain metabolite quinolinic acid. Neurosci Lett.

[CR14] Fuchs D, Forsman A, Hagberg L, Larsson G, Norkrans G, Reignegger G (1990). Immune activation and decreased tryptophan in patients with HIV-1 infection. J Interf Res.

[CR15] Fuchs D, Moller AA, Reibnegger G, Werner ER, Werner-Felmayer G, Dierich MP (1991). Increased endogenous interferon-gamma and neopterin correlate with increased degradation of tryptophan in human immunodeficiency virus type 1 infection. Immunol Lett.

[CR16] Grill M, Gisslen M, Cinque P, Peterson J, Lee E, Spudich S, et al (n.d.) Kynurenine-tryptophan and phenylalanine-tyrosine levels in cerebrospinal fluid in HIV infection. 19th Conference on Retroviruses and Opportunistic Infections, 5–8 March 2012, Seattle, WA, p 463

[CR17] Guillemin GJ, Smith DG, Smythe GA, Armati PJ, Brew BJ (2003). Expression of the kynurenine pathway enzymes in human microglia and macrophages. Adv Exp Med Biol.

[CR18] Guillemin GJ, Smythe G, Takikawa O, Brew BJ (2004). Expression of indoleamine 2,3-dioxygenase and production of quinolinic acid by human microglia, astrocytes, and neurons. Glia.

[CR19] Gunthard HF, Saag MS, Benson CA, del Rio C, Eron JJ, Gallant JE (2016). Antiretroviral drugs for treatment and prevention of HIV infection in adults: 2016 recommendations of the International Antiviral Society-USA Panel. JAMA.

[CR20] Heyes MP, Brew BJ, Saito K, Quearry BJ, Price RW, Lee K, Bhalla RB, der M, Markey SP (1992). Inter-relationships between quinolinic acid, neuroactivity, kynurenines, neopterin and *β*_2_-microglobulin in cerebrospinal fluid and serum of HIV-1-infected patients. J Neuroimmunol.

[CR21] Keegan MR, Chittiprol S, Letendre SL, Winston A, Fuchs D, Boasso A, Iudicello J, Ellis RJ (2016). Tryptophan metabolism and its relationship with depression and cognitive impairment among HIV-infected individuals. Int J Tryptophan Res.

[CR22] Laich A, Neurauter G, Widner B, Fuchs D (2002). More rapid method for simultaneous measurement of tryptophan and kynurenine by HPLC. Clin Chem.

[CR23] Larsson M, Hagberg L, Norkrans G, Forsman A (1989). Indoleamine deficiency in blood and cerebrospinal fluid from patients with human immunodeficiency virus infection. J Neurosci Res.

[CR24] Launay JM, Copel L, Callebert J, Corvaïa N, Lepage E, Bricaire F, Saal F, Peries J (1988). Decreased whole blood 5-hydroxytryptamine (serotonin) in AIDS patients. J Acquir Immune Defic Syndr.

[CR25] McDonagh EM, Lau JL, Alvarellos M, Altman RB, Klein TE (2015). PharmGKB summary: efavirenz pathway, phamacokinetcs (PK). Pharmacogenet Genomics.

[CR26] Murray MF (2007). The human indoleamine 2,3-dioxygenase gene and related human genes. Curr Drug Metab.

[CR27] Robertson K, Liner J, Meeker RB (2012). Antiretroviral neurotoxicity. J Neuro-Oncol.

[CR28] Rowlands J, Higgs C, De Esteban N, McKenna S, Perry N, Winston A, et al (n.d.) Multicenter open-label study of switching from atripla to eviplera for CNS toxicity. 54th Interscience Conference on Antimicrobial Agents and Chemotherapy, September 5–9, 2014. Washington, DC. Abstract H-1005

[CR29] Stone TW (1993). Neuropharmacology of quinolinic and kynurenic acids. Pharmacol Rev.

[CR30] Strasser B, Gostner JM, Fuchs D (2016). Mood, food and cognition: role of tryptophan and serotonin. Curr Opin Clin Nutr Metab Care.

[CR31] Summary of product characteristics, Sustiva®. Last updated on eMC 01-Nov-2016

[CR32] Tovar-y-Romo LB, Bumpus NN, Pomerantz D, Avery LB, Sacktor N, McArthur JC, Haughey NJ (2012). Dendritic spine injury induced by the 8-hydroxy metabolite of efavirenz. J Pharmacol Exp Ther.

[CR33] Underwood J, Robertson KR, Winston A (2015). Could antiretroviral neurotoxicity play a role in the pathogenesis of cognitive impairment in treated HIV disease?. AIDS.

[CR34] United States Department of Health & Human Services (DHHS) (n.d.) Guidelines for the use of antiretroviral agents in HIV-1-infected adults and adolescents. 14th July 2016. http://aidsinfo.nih.gov/guidelines

[CR35] Waters L, Fisher M, Winston A, Higgs C, Hadley W, Garvey L, Mandalia S, Perry N, Nicola M, Nelson M (2011). A phase IV, double-blind, multicentre, randomized, placebo-controlled, pilot study to assess the feasibility of switching individuals receiving efavirenz with continuing central nervous system adverse events to etravirine. AIDS.

[CR36] Widner B, Werner ER, Schennach H, Wachter H, Fuchs D (1997). Simultaneous measurement of serum tryptophan and kynurenine by HPLC. Clin Chem.

[CR37] Yapa HM, Waters L, Rowlands J, Jackson A, Madndalia S, Higgs C, et al (n.d.) The feasibility of switching efavirenz (EFV)-based highly active antiretroviral therapy to raltegravir (RAL)-based therapy in HIV-infected individuals with central nervous system (CNS) toxicity: a phase IV open label pilot study. 7th International AIDS Society Conference on HIV Pathogenesis, Treatment and Prevention, June 30–July 3, 2013, Kuala Lumpur. Abstract MOPE090

[CR38] Zangerle R, Widner B, Quirchmair G, Neurauter G, Sarcletti M, Fuchs D (2002). Effective antiretroviral therapy reduces degradation of tryptophan in patients with HIV-1 infection. Clin Immunol.

[CR39] Zheve GT (2007). Neuroprotective mechanisms of nevirapine and efavirenz in a model of neurodegeneration.

